# The impact of contour variation on tumour control probability in anal cancer

**DOI:** 10.1186/s13014-018-1033-y

**Published:** 2018-05-18

**Authors:** Michael P. Jones, Jarad Martin, Kerwyn Foo, Patrick Estoesta, Lois Holloway, Michael Jameson

**Affiliations:** 1 School of Medicine and Public Health, University of Newcastle, Callaghan, NSW Australia; 2 Department of Radiation Oncology, Calvary Mater Newcastle, Waratah, NSW Australia; 3 Department of Radiation Oncology, Chris O’Brien Lifehouse, Camperdown, NSW Australia; 4 Liverpool and Macarthur Cancer Therapy Centres, Liverpool, NSW Australia; 5 Ingham Institute for Applied Medical Research, Liverpool, NSW Australia; 6 South West Clinical School, Faculty of Medicine, University of New South Wales, Kensington, NSW Australia; 7 Institute of Medical Physics, School of Physics, University of Sydney, Camperdown, NSW Australia; 8 Centre for Medical Radiation Physics, University of Wollongong, Wollongong, NSW Australia

**Keywords:** Anal cancer, Chemo-radiotherapy, Squamous cell carcinoma, Tumour control probability

## Abstract

**Background:**

While intensity modulated radiotherapy (IMRT) has been widely adopted for the treatment of anal cancer (AC), the added contour complexity poses potential risks. This study investigates the impact of contour variation on tumour control probability (TCP) when using IMRT for AC.

**Methods:**

Nine Australian centres contoured a single computed tomography dataset of a patient with AC. The same optimised template-based IMRT planning protocol was applied to each contour set to generate nine representative treatment plans and their corresponding dose volume histograms. A geometric analysis was performed on all contours. The TCP was calculated for each plan using the linear quadratic and logitEUD model.

**Results:**

The median concordance index (CI) for the bladder, head and neck of femur, bone marrow, small bowel and external genitalia was 0.94, 0.88, 0.84, 0.65 and 0.65, respectively. The median CI for the involved nodal, primary tumour and elective clinical target volumes were 0.85, 0.77 and 0.71, respectively. Across the nine plans, the TCP was not significantly different. Variation in TCP between plans increased as tumour cell load increased or radiation dose decreased.

**Conclusions:**

When using IMRT for AC, contour variations generated from a common protocol within the limits of minor deviations do not appear to have a significant impact on TCP. Contouring variations may be more critical with increasing tumour cell load or reducing radiotherapy dose.

## Background

Squamous cell carcinoma (SCC) of the anal canal (AC) is an uncommon tumour with an increasing incidence [1–3]. The treatment standard is organ sparing chemo-radiotherapy (CRT) [4, 5], which achieves loco-regional control in 63–86% [6–8] of patients but carries a risk of significant acute and late toxicity [9].

In RTOG 0529, Intensity Modulated Radiotherapy (IMRT) was shown to reduce acute toxicity and cause fewer treatment interruptions compared with historical controls [10]. It also highlighted the increased complexity of IMRT. Remarkably, 81% of plans required revision and 46% required multiple revisions. Despite the provision of a contouring atlas, most revisions were required for incorrect contouring.

Contouring errors in IMRT based CRT increase loco-regional recurrence in head and neck SCC [11, 12]. This raises concerns for AC patients treated with IMRT by inexperienced clinicians. Indeed, population based data suggests AC patients treated at high volume centres have improved survival [13].

The Trans-Tasman Radiation Oncology Group (TROG) investigated IMRT as a new technology in the treatment of AC as part of their Assessment of New Radiation Oncology Technology and Treatments (ANROTAT) [14]. Their study compared the safety, clinical efficacy and cost effectiveness of IMRT with three-dimensional conformal radiation therapy (3DCRT).

The credentialing component of this study involved a benchmarking assessment performed by nine Australian centres. Each centre was provided a computed tomography (CT) dataset of a female patient with T3 N2 AC (primary greater than 50 mm with involved mesorectal and inguinal lymph nodes [LN]) to be treated with 54Gy in 30 fractions. Centres were asked to contour clinical target volumes (CTV) and organs at risk (OAR).

Having acquired these contours from TROG, we sought to describe CTV and OAR contour variability as well as model the impact of contour variation on tumour control probability (TCP) when using IMRT for AC.

## Methods

### Treatment planning

Each of the nine Australian centres participating in the TROG TRP11.A (ANROTAT) Study B contoured OAR and CTVs using a standard protocol (Table 1) and the Australian Gastrointestinal Trials Group (AGITG) contouring atlas [15].

**Table 1:** Tumour and organ at risk contoured volumes

Tumour Volumes	Organs at Risk
Primary clinical target volume to receive 54Gy (CTV54p)	Small bowel
Nodal clinical targe volume to receive 54Gy (CTV54n)	Bladder
Elective clinical target volume to receive 45Gy (CTV45)	External genitalia
Note: CTV45 included mesorectal, pre-sacral, internal iliac, external iliac, obturator and inguinal lymph nodes	Head and neck of femur Bone marrow (ilium)

Varian’s Eclipse treatment planning system was used to generate nine representative IMRT plans. An optimised template-based IMRT planning protocol was initially applied to each contour set. If dosimetric parameters were not met in all plans, the template was adjusted and re-applied to all plans until all target volume and OAR constraints were met. Planning Target Volume (PTV) coverage requirements and OAR constraints were based on ANROTAT guidelines (Appendix).

### Geometric analysis

All observer generated contours were collated on a single copy of the benchmarking CT dataset. A reference contour was generated using the STAPLE [16] algorithm in the Computational Environment for Radiotherapy Research (CERR) [17]. In-house code was used to analyse the geometric variation in contours. Metrics of comparison included two volume overlap indices; concordance index (CI) and dice similarity coefficient (DSC).

### Tumour control probability

**Fig. 1:**
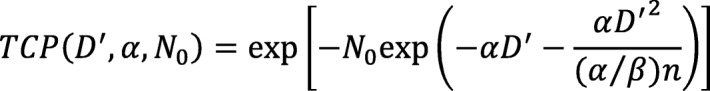
The linear quadratic model

**Fig. 2:**
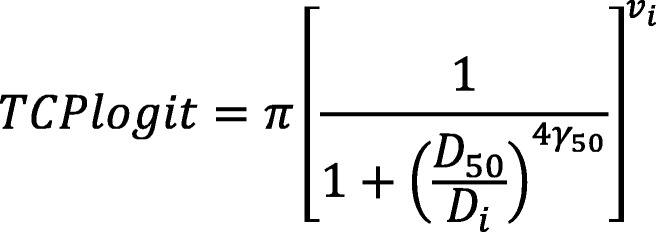
The logitEUD model

Dose volume histogram (DVH) data was extracted from the treatment plans using CERR [17] and Matlab (The Mathworks, Natick, USA). From the DVHs, CompPlan [18] (open source software) calculated a TCP for each plan using a linear quadratic and logitEUD model (shown in Figs. 1 and 2). The models included a fraction-size effect correction.

For the linear quadratic model where n is the number of fractions and D’ is the total dose, an α/β ratio of 10Gy, α of 0.196 and N_0_ (number of initial clonogens) of 34,900 were used [19]. A sensitivity analysis investigated the effect on TCP of increasing N_0_ and decreasing prescription dose.

For the logitEUD model where v_i_ is a voxel, γ_50_ is the slope of the dose response curve at D_50_ and D_i_ is the total dose delivered to each voxel, D_50_ values (dose required for a 50% probability of tumor control at 2 years) for gross and microscopic disease were 42Gy and 30Gy, respectively [19]. Given these values were taken from curves fit to patients treated with CRT, the effect of concurrent chemotherapy was implicitly incorporated.

## Results

### Geometric analysis

Table 2 shows the results of the OAR geometric analysis. There was close contour agreement for the bladder (median concordance index [CI] 0.94), head and neck of femur (CI 0.88) and bone marrow (CI 0.84). Greater contour variation was seen in the small bowel (CI 0.65) and external genitalia (CI 0.65).

**Table 2:** Organ at risk geometric analysis

Organs at Risk	Stats	CI	DSC	Volume (cm3)
Small bowel	Median	0.65	0.79	543.22
	Range	0.12	0.10	132.37
Bladder	Median	0.94	0.97	156.37
	SD	0.01	0.01	6.43
External genitalia	Median	0.65	0.79	130.61
	SD	0.19	0.17	65.47
Head and neck of femur	Median	0.88	0.93	202.29
	SD	0.06	0.03	23.73
Bone marrow (ilium)	Median	0.84	0.91	371.61
	SD	0.06	0.03	22.62

*SD*: standard deviation, *CI*: concordance index, *DSC*: dice similarity coefficient

**Fig. 3:**
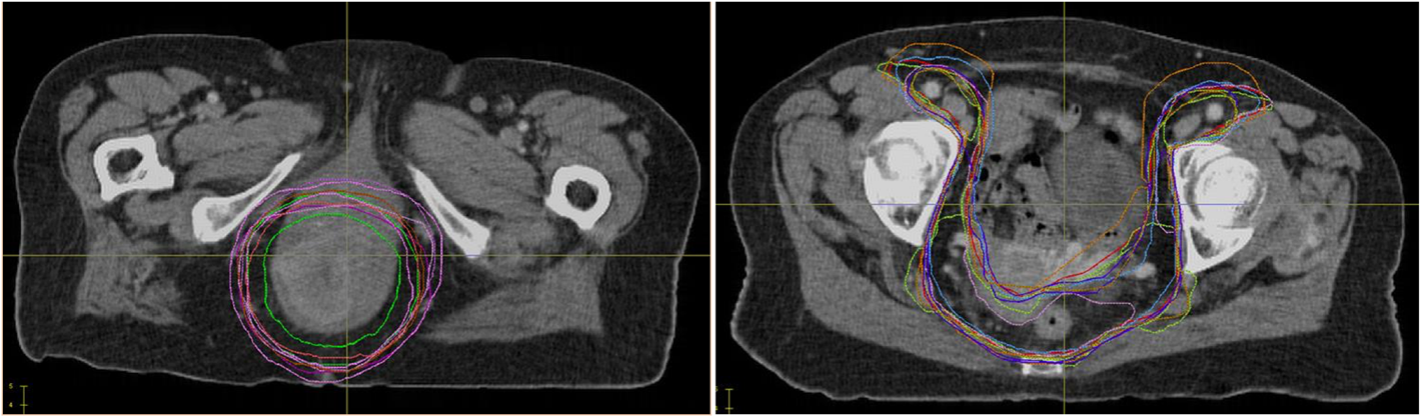
Tumour volumes contoured by the nine participating centres. Left: Primary clinical target volumes (CTV54p), Right: Elective clinical targe volumes (CTV45)

Table 3 shows the results of the tumour volume geometric analysis. Increasing contour variation was seen in CTV54n (CI 0.85), CTV54p (CI 0.77) and CTV45 (CI 0.71), respectively. The CTV45 and CTV54p contours are shown in Fig. 3.

**Table 3:** Tumour control probability and tumour volume geometric analysis

Volume	Stats	TCP (LQ)	TCP (Logit)	CI	DSC	Volume (cm3)
CTV45	Median	0.84	0.60	0.71	0.82	1299.38
	SD	0.18	0.04	0.13	0.10	190.64
CTV54p	Median	0.94	0.52	0.77	0.86	458.83
	SD	0.11	0.02	0.17	0.13	132.92
CTV54n	Median	1.00	0.53	0.85	0.89	37.92
	SD	0.00	0.02	0.26	0.21	7.69

*CTV45*: elective clinical targe volume, *CTV54p*: primary clinical target volume, *CTV54n*: nodal clinical target volume, *SD*: standard deviation, *CI*: concordance index, *DSC*: dice similarity coefficient

### Tumour control probability

The TCP for CTVs are shown in Table 3. Using the linear quadratic model, the median TCP for CTV45, CTV54p and CTV54n was 0.84 (standard deviation [SD] 0.18), 0.94 (SD 0.11) and 1.00 (SD 0.00), respectively. Using the logitEUD model, the median TCP for CTV45, CTV54p and CTV54n was 0.60 (SD 0.04), 0.52 (SD 0.02) and 0.53 (SD 0.02).

**Fig. 4:**
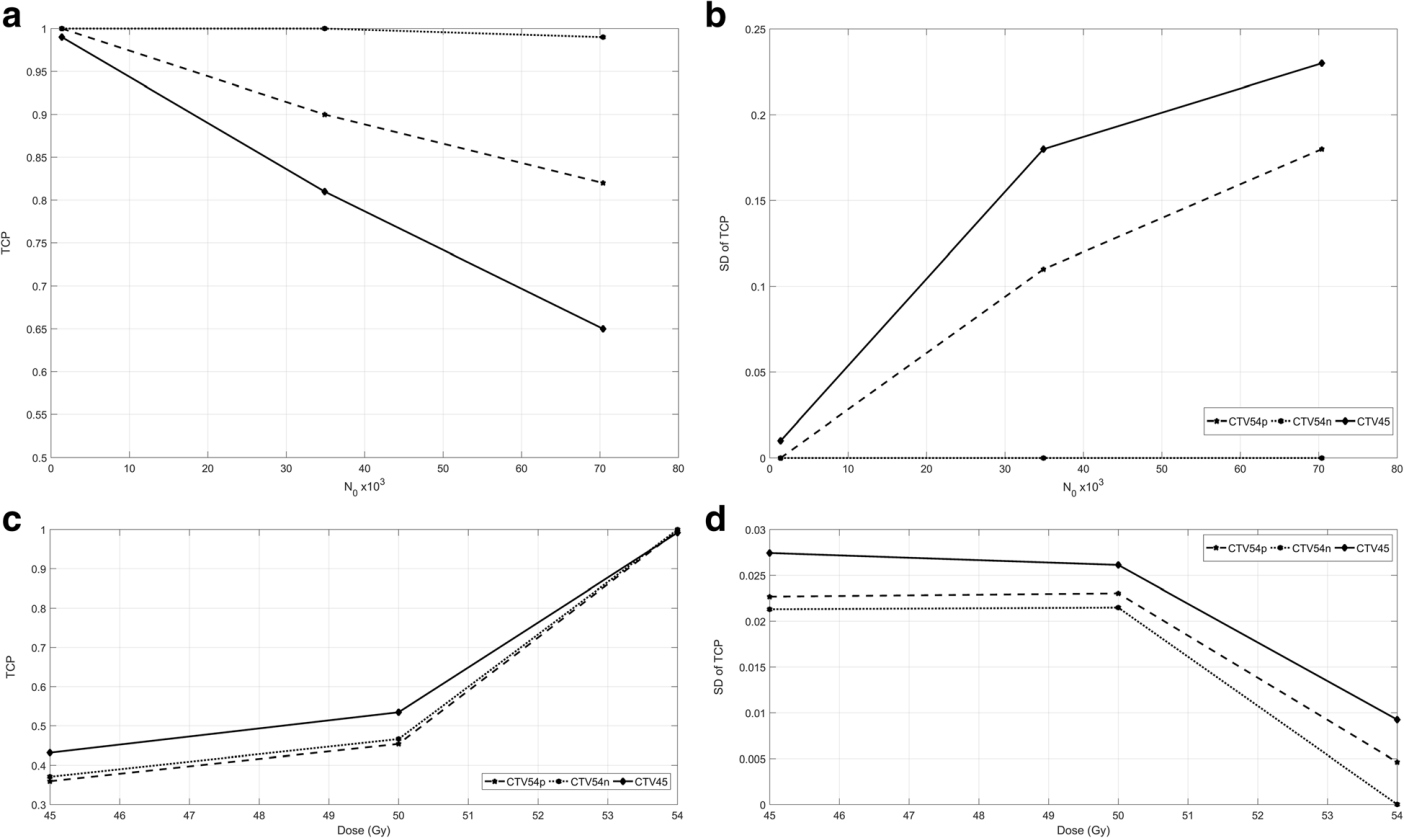
Sensitivity analyses. **a**: Tumour control probability with changing N_0_, **b**: variation in tumour control probability between plans with changing N_0_, **c**: tumour control probability with changing dose, **d**: variation in tumour control probability between plans with changing dose. TCP = Tumour Control Probability, SD = Standard Deviation, N_0_ = number of initial clonogens, Gy = Gray, CTV54p = Primary clinical target volume to receive 54Gy, CTV54n = Nodal clinical targe volume to receive 54Gy, CTV45 = Elective clinical target volume to receive 45Gy

Sensitivity analysis of the linear quadratic model revealed that the TCP reduced as tumour cell load increased (Fig. 4a) but variation in TCP between plans increased (Fig. 4b). Likewise, as radiation dose decreased, TCP reduced (Fig. 4c) and variation in TCP between plans increased (Fig. 4d).

## Discussion

### Organ at risk contour variation

Our results reveal while there was good inter-observer agreement for the bladder (CI: 0.94), head and neck of femur (CI 0.88) and bone marrow (CI: 0.84), there was significant contour variation in the small bowel (CI 0.65) and external genitalia (CI: 0.65).

For the small bowel, inclusion of the large bowel, iliac vessels, ovaries, peritoneum and muscle were common contouring errors. The lack of oral contrast may have contributed to these inaccuracies. Oral contrast delivered 1 h prior to CT simulation is usually recommended to improve small bowel identification [20].

Controversy persists regarding the optimal bowel contouring technique. No consensus was reached by RTOG [20] and QUANTEC [21] list dose constraints for both bowel loops and a bowel bag. While a bowel bag accounts for the mobility of viscera and simplifies OAR contouring, it may have limitations for post-surgical patients and in capturing inferior motion [22].

Misidentification of the small (60%) and large (45%) bowel was also common in plans submitted to RTOG 0529 [23]. However, accuracy of the external genitalia contours was not reported. In fact the RTOG pelvic OAR contouring guidelines do not include instructions for contouring external genitalia [20] and no consensus guidelines exist. While United Kingdom guidelines have been proposed [24], until consensus guidelines are formalised, variation in external genitalia contours is likely to persist.

For the development and implementation of evidenced based OAR dose constraints, consistent contouring is imperative [25, 26]. This is particularly true in AC IMRT where the proximity of OAR and tumour volumes often requires a compromise between adequate coverage and safety.

### Tumour volume contour variation

Despite being provided gross tumour volumes (GTV), variations in the protocoled CTV expansions were seen: CTV54p (CI 0.77), CTV54n (CI 0.85) and CTV45 (CI 0.71). Unsurprisingly, the greatest variation was seen in the CTV45 volumes. These are the largest and the least influenced by the GTV. Incorrect elective nodal contouring was also common in RTOG 0529 where errors were seen in the mesorectum (55%), pre-sacral (43%), inguinal (33%) and iliac nodal groups (31%).

The greatest discrepancies in the CTV45 volumes were in the anterior direction with variable expansion into the uterus and bladder. The AGITG guidelines recommend an internal margin expansion of 1 cm into the bladder and uterus to account for bladder volume variation [15].

The greatest discrepancies in the CTV54p volumes were also in the anterior direction with variable expansion into the vagina and genitalia. Some have highlighted the risk of genital relapse when IMRT is used to achieve genital sparing [27]. While this has been challenged more recently [28], we emphasise the priority of adequate tumour coverage over genital sparing and stress the importance of accurate multi-modality baseline imaging to help optimally define the extent of disease.

Contouring atlases for anorectal volumes have been shown to reduce inter-observer variability [29], however, atlases were available to observers in both the RTOG and TROG studies. Perhaps this variation can be attributed to clinicians coming to terms with a new technique.

A literature review identified guidelines, teaching, auto-contouring and multi-modality imaging as interventions that reduce inter-observer variability in contour delineation [30]. Online teaching and contouring workshops are now offered by international cancer societies and clinicians can have their volumes peer reviewed at their centre and through participation in clinical trials incorporating radiotherapy quality assurance.

### Tumour control probability

IMRT is the treatment of choice for AC [31]. It enhances the therapeutic window by reducing dose to OAR and resultant acute toxicity [23]. Fewer treatment breaks for toxicity are required and it offers the prospect of safe dose escalation for large tumours. However, AC contouring is complex and considerable variation exists in target volume delineation [29, 31].

We have shown that minor contour variations generated from a common protocol do not translate into significant differences in TCP. By applying a template-based IMRT planning protocol, we isolated the impact of contour variation on TCP. Further TCP variability would be expected should centres develop their own treatment plans or employ different treatment doses.

Contours for this study were generated from a common contouring guideline [14]. Contouring guidelines lead to greater inter-observer agreement [32] and the provision of an anorectal contouring atlas has been shown to increase TCP and reduce normal tissue complication probability (NTCP) [29].

GTV contours were supplied in the TROG study which may have reduced CTV variation. Inaccurate GTV delineation occurred in 21% of plans submitted to RTOG 0529 [23]. Target volume delineation is more accurate with the use of co-registered Positron Emission Tomography (PET) and Magnetic Resonance Imaging (MRI) and is recommended for treatment planning [31]. PET is recommended to identify lymph nodes requiring inclusion in the GTV [33].

### Sensitivity analysis

We investigated the influence of prescription dose and N_0_ (number of initial clonogens) on the TCP predicted by the linear quadratic model. Increasing N_0_ led to a reduction in TCP (Fig. 4a) and an increase in the TCP variation between plans (Fig. 4b). Decreasing dose led to a reduction in TCP (Fig. 4c) and increase in TCP variation between plans (Fig. 4d).

While the influence of dose and N_0_ on the absolute TCP is not surprising, the effect on TCP variation is quite interesting. It suggests contour variations become more critical as one moves down the dose response curve either by increasing tumour cell load or reducing dose. These are important insights for radiation dose de-escalation trials such as ACT 3 and 4 [34] and highlights the central role real-time radiotherapy quality assurance should play in these studies.

### Limitations and future directions

In radiation therapy, TCP models have traditionally been limited to the relative comparison of treatment plans rather than their absolute predictive power. Indeed, the difference in tumour control estimated by the two models used in our study highlights the dangers of over interpreting TCP results or using them to inform radiotherapy dose selection.

That contours were completed in the context of a trial using a common protocol with a GTV already provided is a shortcoming of this study potentially limiting its applicability to the wider community. Our results are likely to underestimate contour variation and do not capture the effect of radiotherapy plan generation.

Our study aim was to isolate the impact of contour variation on TCP. While we appreciate there is manual bias in planning [35], it is out of the scope of this study. We tried to mitigate against this by using a single planner and single patient anatomy, deploying a class solution beam arrangement, objectives and priorities.

While a NTCP assessment was explored, this was abandoned due to a lack of reliable radiobiological parameters in the literature. The principle benefit of IMRT is reduced OAR dose, avoidance of toxicity induced treatment gaps and the prospect of safe dose escalation for larger tumours. To achieve this, tumour volumes must balance adequate coverage of at risk sites with toxicity risk.

The ACT 3–5 [34] studies will provide valuable insights into the radiation dose response curve for AC. The incorporation of prognostic factors other than stage such as human papilloma virus status could help further refine this relationship [36]. Treatment adaptation represents another promising approach and may ultimately provide the best means of individualising treatment [37].

## Conclusions

In the context of a clinical study, with the dose and parameters used, minor contour variations generated from a common guideline did not significantly impact TCP. With increasing tumour cell load or reducing RT dose, contour variations may be more critical.

## Appendix

**Table:**

Volume	Constraint
PTV 45	D95 ≥ 40.5Gy
	D98 ≥ 36Gy
	D98 ≥ 36Gy
PTV 54	D95 ≥ 48.6
	D98 ≥ 43.2Gy
	D2% ≤ 62.1Gy
Small bowel	V30Gy ≤ 200 cm^3^
	V35Gy ≤ 150 cm^3^
	V45Gy ≤ 20 cm^3^
	V50Gy = 0 cm^3^
Femoral heads	V30Gy ≤ 50%
	V40Gy ≤ 35%
	V44Gy ≤ 5%
Iliac crest	V30Gy ≤ 50%
	V40Gy ≤ 35%
	V50Gy ≤ 5%
External genitalia	V20Gy ≤ 50%
	V30Gy ≤ 35%
	V40Gy ≤ 5%
Bladder	V35Gy ≤ 50%
	V40Gy ≤ 35%
	V50Gy ≤ 5%

## Abbreviations

3DCRT: Three-dimensional conformal radiation therapy
AC: Anal cancer
AGITG: Australian Gastrointestinal Trials Group
ANROTAT: Assessment of New Radiation Oncology Technology and Treatments
CERR: Computational Environment for Radiotherapy Research
CI: Concordance Index
CRT: Chemo-radiotherapy
CT: Computed tomography
CTV: Clinical target volume
CTV45: Elective clinical target volume to receive 45Gy
CTV54n: Nodal clinical target volume to receive 54Gy
CTV54p: Primary clinical target volume to receive 54Gy
DSC: Dice similarity coefficient,
DVH: Dose Volume Histogram
GTV: Gross tumour volumes,
IMRT: Intensity modulated radiotherapy
LN: Lymph node
MRI: Magnetic Resonance Imaging
OAR: Organs at risk
PET: Positron Emission Tomography
PTV: Planning Target Volume
SCC: Squamous cell carcinoma
SD: Standard Deviation
TCP: Tumour Control Probability
TROG: Trans-Tasman Radiation Oncology Group
